# Glucagon-like peptide-1 receptor agonists in patients with heart failure with reduced ejection fraction

**DOI:** 10.1093/eschf/xvag111

**Published:** 2026-04-17

**Authors:** Joseph Kassab, Mark H Drazner, Jennifer T Thibodeau

**Affiliations:** Department of Internal Medicine, UT Southwestern Medical Center, Dallas, TX, USA; Division of Cardiology, UT Southwestern Medical Center, 5323 Harry Hines Blvd, Dallas, TX 75390, USA; Division of Cardiology, UT Southwestern Medical Center, 5323 Harry Hines Blvd, Dallas, TX 75390, USA

**Keywords:** GLP-1 RA, HFrEF, Semaglutide, Tirzepatide, Liraglutide

## Abstract

**Background and Aims:**

Glucagon-like peptide-1 receptor agonists (GLP-1RAs) improve cardiovascular outcomes in obesity and heart failure and preserved ejection fraction; however, their safety and efficacy in heart failure with reduced ejection fraction (HFrEF) remain uncertain. We sought to evaluate the efficacy and safety of GLP-1RAs in patients with HFrEF.

**Methods:**

We conducted a multicentre retrospective cohort study of adult patients with HFrEF (LVEF ≤40%) between 2021 and 2024. Patients receiving semaglutide, tirzepatide, or liraglutide were compared with GLP-1RA-naïve patients. Propensity score matching (PSM) (1:1; caliper 0.1) was conducted to balance demographics, comorbidities, LVEF, body mass index (BMI), haemoglobin A1c (HbA1c), and guideline-directed medical therapy. Primary outcomes were 1-year all-cause mortality and acute decompensated HF (ADHF) hospitalizations. Secondary outcomes included new acute coronary syndrome (ACS), stroke/transient ischaemic attack (TIA), atrial fibrillation (AF)/flutter, and ventricular tachycardia (VT)/ventricular fibrillation (VF) events.

**Results:**

A total of 127 021 patients met inclusion criteria. After PSM, 2550 patients (*n* = 1275 per group) were analysed (mean age 61.5 ± 13 years, 33.5% female, 66% White, mean HbA1c 8.1 ± 2.1%, mean LVEF 30 ± 8.7%, mean BMI 34.5 ± 8.4 kg/m^2^). Patients prescribed GLP-1RAs had a lower risk of all-cause mortality [7.1% vs 10.2%, odds ratio (OR): 0.68, 95% confidence interval (CI): 0.51–0.90; *P* = .006]. Time-to-event analysis was also consistent [matched hazard ratio: 0.54 (95% CI: 0.41–0.7); *P* < .0001]. Patients in the GLP-1RA group also had a lower risk of ADHF [27.7% vs 32.8% OR: 0.79 (95% CI: 0.66–0.93); *P* = .005]. New onset ACS, stroke/TIA, AF/flutter, and VT/VF events were similar in both groups.

**Conclusions:**

In this real-world cohort, GLP-1RA therapy in HFrEF was associated with reduced mortality and ADHF without an increase in arrhythmic events. Prospective randomized trials are needed to validate these findings.

## Graphical Abstract

**Graphical Abstract:**
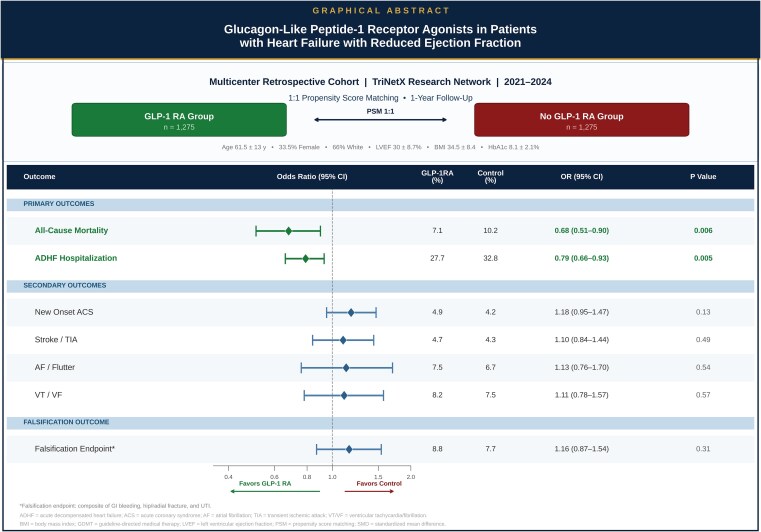

## Background

Glucagon-like peptide-1 receptor agonists (GLP-1RAs) are promising therapeutic agents in patients with established cardiovascular disease and obesity^1^ and in patients with heart failure and preserved ejection fraction (HFpEF),^2,3^ yet their utility in heart failure with reduced ejection fraction (HFrEF) remains uncertain. Prior trials, including FIGHT^4^ (liraglutide in advanced HFrEF) and LIVE^5^ (liraglutide in stable chronic HFrEF), reported conflicting findings, possibly due to markedly reduced left-ventricular ejection fraction (LVEF) (mean ∼25% in FIGHT) or lack of significant obesity (mean body massr index [BMI] ∼28 kg/m^2^ in LIVE). Moreover, the obesity–heart failure paradox, wherein patients with higher BMI have been observed to experience better survival in HFrEF compared with their leaner counterparts, raises concern regarding the safety and long-term implications of intentional weight loss in this population.^6^

## Aims

We sought to evaluate the efficacy and safety of GLP-1RAs in patients with HFrEF.

## Methods

We conducted a retrospective cohort analysis of de-identified, aggregated patient data from the TriNetX research network, which contains data from the electronic health records of ∼115 million patients from 72 healthcare organizations (HCOs), both in the inpatient and outpatient settings, primarily in the USA. Patients aged ≥18 years with HFrEF as confirmed by transthoracic echocardiography (TTE) with LVEF ≤40%, on guideline-directed medical therapy (GDMT), were identified between January 2021 and December 2024. They were divided into two groups based on GLP-1RA use: (i) patients never prescribed GLP-1RAs, and (ii) patients with semaglutide, tirzepatide, or liraglutide initiation 3 months before or after index TTE. The primary outcomes were all-cause mortality and acute decompensated HF (ADHF) exacerbations leading to inpatient admission (defined by ICD-10 codes and need for IV diuretics). Secondary outcomes were new onset acute coronary syndrome (ACS), new onset stroke or transient ischaemic attack (TIA), new onset atrial fibrillation (AF)/flutter, and new ventricular tachycardia (VT)/fibrillation (VF) events. Outcomes were analysed over a 1-year follow-up period. Falsification outcomes included a composite incidence of gastrointestinal (GI) bleeding, hip or radial fracture, and urinary tract infection (UTI) during the follow-up period.

Covariates [notably baseline demographics, BMI, major comorbidities, prescribed medications including GDMT, presence or absence of an implantable cardioverter defibrillator (ICD), LVEF, *N*-terminal pro-B-type natriuretic peptide (NT-proBNP) levels, estimated glomerular filtration rate (eGFR), and haemoglobin A1c (HbA1c)] were matched by 1:1 propensity score matching (PSM) using the greedy nearest-neighbour algorithm with a caliper of 0.1 pooled standardized mean difference (SMD). Any characteristic with a SMD <0.1 between the two groups was considered well-matched. The measures of association included odds ratios (ORs) on the matched population for primary and secondary outcomes. Survival analyses were performed for each outcome by plotting Kaplan–Meier curves with log-rank tests; additionally, Cox proportional hazard models were used to calculate the hazard ratio (aHR) to compare the two groups after PSM. A *P*-value of *<*.05 was considered statistically significant. Analyses were conducted using the embedded R model within the TriNetX platform. Institutional Review Board approval was exempted by the University of Texas Southwestern Review Board, as the study utilized aggregate, de-identified data from a research network database.

## Results

A total of 127 021 patients met inclusion criteria. At baseline, patients who were prescribed GLP-1RAs (*n* = 1310) were younger (mean age 61.2 ± 13 vs 68.8 ± 14.3 years) and had higher baseline BMI (34.9 ±8.9 vs 28.6 ± 7.1 kg/m^2^), higher LVEF (30.2 ± 8.8% vs 28.3 ± 9.1%), and higher HbA1c (8.2 ± 2.2% vs 6.7 ± 1.8%). After PSM, the matched cohort consisted of 2550 patients (*n* = 1275 patients per group). The majority of covariates were well balanced (SMD <0.1) (mean age 61.5 ± 13 years, 34% female, 66% White, mean HbA1c 8.1 ± 2.1%, mean LVEF 30 ± 8.7%, mean BMI 34.5 ± 8.4 kg/m^2^) (*Table 1*). Guideline-directed medical therapy was balanced across both groups, with 95% of the cohort on a beta-blocker, 85% on an angiotensin-converting enzyme inhibitor (ACEi), angiotensin receptor blocker (ARB), or angiotensin receptor–neprilysin inhibitor (ARNI), 43% on a mineralocorticoid receptor antagonist (MRA), and 48% on a sodium–glucose cotransporter-2 inhibitor (SGLT2 inhibitor). In the GLP-1RA group, 70% were on semaglutide (2.4 mg subcutaneously weekly), 12.8% were on tirzepatide (10–15 mg subcutaneously weekly), and 17.2% were on liraglutide (3.0 mg subcutaneously daily). Median duration on a GLP-1RA was 287 days.

**Table 1:** Patient baseline characteristics before and after propensity-score matching

Characteristics	GLP-1RA (*N* = 1310)	No GLP-1RA (*N* = 125 711)	SMD (pre-match)	GLP-1RA (*N* = 1275)	No GLP-1RA (*N* = 1275)	SMD (post-match)
**Demographics**						
Age at index (years)	61.2 ± 13	68.8 ± 14.3	0.56	61.5 ± 12.8	61.5 ± 14.1	**0**.**01**
Female (%)	434 (33.1)	40 394 (32.1)	0.02	422 (33.1)	446 (35)	**0**.**03**
White (%)	872 (66.6)	89 854 (71.5)	0.11	850 (66.7)	831 (65.2)	**0**.**03**
Black (%)	325 (24.8)	27 583 (21.9)	0.01	317 (24.9)	325 (25.5)	**0**.**01**
Hispanic (%)	215 (16.4)	14 223 (11.3)	0.15	211 (16.5)	220 (17.3)	**0**.**02**
BMI (kg/m^2^)	34.9 ± 8.9	28.6 ± 7.11	0.79	34.9 ± 8.91	34.1 ± 7.92	**0**.**09**
**Comorbidities**						
Hypertension (%)	1175 (89.7)	98 642 (78.5)	0.31	1144 (89.7)	1162 (91.1)	**0**.**05**
Hyperlipidaemia (%)	869 (66.3)	60 693 (48.3)	0.37	842 (66)	845 (66.3)	**0**.**01**
AFib/aflutter (%)	393 (30)	38 658 (30.8)	0.02	379 (29.7)	354 (27.8)	**0**.**04**
Diabetes (%)	1161 (88.6)	49 317 (39.2)	1.19	1126 (88.3)	1157 (90.7)	**0**.**07**
Coronary artery disease (%)	886 (67.6)	72 614 (57.8)	0.21	867 (68)	863 (67.7)	**0**.**01**
Chronic kidney disease (%)	567 (43.3)	45 217 (36)	0.15	738 (57.9)	725 (56.9)	**0**.**01**
Chronic obstructive lung disease (%)	224 (17.1)	25 673 (20.4)	0.09	219 (17.2)	218 (17.1)	**0**.**002**
Neoplasm (%)	196 (15)	21 523 (17.1)	0.06	190 (14.9)	201 (15.8)	**0**.**02**
Cerebrovascular accident (%)	122 (9.3)	10 559 (8.4)	0.03	119 (9.3)	134 (10.5)	**0**.**04**
Peripheral artery disease (%)	110 (8.4)	10 937 (8.7)	0.01	111 (8.7)	115 (9.0)	**0**.**01**
Implantable cardioverter defibrillator (%)	377 (28.8)	20 587 (16.4)	0.30	354 (27.8)	342 (26.8)	**0**.**02**
Left bundle branch block (%)	211 (16.1)	14 130 (11.2)	0.14	205 (16.1)	189 (14.8)	**0**.**03**
Cardiac resynchronization therapy (CRT-D)	92 (7)	5028 (4)	0.13	89 (7)	77 (6)	**0**.**04**
Diastolic dysfunction (>Grade 3) (%)	200 (15.3)	15 061 (12)	0.09	195 (15.3)	186 (14.6)	**0**.**02**
**Metrics**						
LVEF (%)	30.2 ± 8.8	28.3 ± 9.1	0.22	30.4 ± 8.8	29.6 ± 8.6	**0**.**09**
HbA1c (%)	8.2 ± 2.2	6.7 ± 1.8	0.78	8.2 ± 2.2	8.0 ± 2	**0**.**09**
Haemoglobin (g/dL)	12.5 ± 2.4	11.9 ± 2.4	0.25	12.5 ± 2.4	12.1 ± 2.5	**0**.**17**
Serum creatinine (mg/dL)	1.5 ± 1.3	1.6 ± 1.5	0.08	1.5 ± 1.3	1.5 ± 1.3	**0**.**02**
NT-proBNP >3000 pg/mL (%)	252 (19.2)	30 548 (24.3)	0.12	244 (19.1)	270 (21.2)	**0**.**05**
eGFR (mL/min/1.73 m^2^)	64.8 ± 31.8	60.1 ± 32.4	0.15	64.3 ± 31.7	64.7 ± 32.5	**0**.**01**
LDL cholesterol (mg/dL)	80.5 ± 41.3	82.8 ± 39	0.05	80.5 ± 41.3	81.6 ± 43.7	**0**.**02**
**Prescribed medications**						
Beta-blocker (%)	1277 (97.5)	92 871 (73.9)	0.55	1236 (96.9)	1216 (95.4)	**0**.**08**
ACE/ARB/ARNi (%) ARNi **(%)**	1117 (85.3)	65 021 (51.7)	0.71	1081 (84.8)	1085 (85.1)	**0**.**01**
	520 (39.7)	9051 (7.2)	0.83	479 (37.6)	459 (36)	**0**.**03**
SGLT2i (%)	677 (51.7)	25 142 (20)	0.43	626 (49.1)	597 (46.8)	**0**.**04**
MRA (%)	594 (45.3)	21 587 (17.2)	0.61	564 (44.2)	551 (43.2)	**0**.**02**
Digoxin (%)	197 (15)	12 570 (10)	0.15	144 (11.3)	140 (11)	**0**.**01**
Loop diuretics (%)	996 (76.0)	92 692 (73.7)	0.04	967 (75.9)	988 (77.5)	**0**.**03**
Statins (%)	1025 (78.3)	94 283 (75)	0.08	997 (78.2)	974 (76.4)	**0**.**04**

ACE, angiotensin-converting enzyme; AFib, atrial fibrillation; ARB, angiotensin II receptor blocker; ARNi, angiotensin receptor–neprilysin inhibitor; BMI, body mass index; CKD, chronic kidney disease; eGFR, estimated glomerular filtration rate; GLP-1RA, glucagon-like peptide-1 receptor agonist; HbA1c, haemoglobin A1c; LVEF, left ventricular ejection fraction; MRA, mineralocorticoid receptor antagonist; PSM, propensity-score matching; SGLT2i, sodium–glucose cotransporter-2 inhibitor; SMD, standardized mean difference (bolded when <0.1).

At 1 year, after PSM, patients prescribed GLP-1RAs had a lower risk of all-cause mortality [7.1% vs 10.2% OR: 0.68, 95% confidence interval (CI): 0.51–0.90; *P* = .006]. Time-to-event analysis was also consistent [matched HR: 0.54 (95% CI: 0.41–0.7); *P* < 0.0001 by log-rank test]. Patients in the GLP-1RA group also had a lower risk of ADHF [27.7% vs 32.8% OR: 0.79 (95% CI: 0.66–0.93); *P* = .005]. New onset ACS was similar in both groups [4.9% vs 4.2% OR: 1.18 (95% CI: 0.95–1.47); *P* = .13] as well as new onset stroke/TIA [4.7% vs 4.3% OR: 1.10 (95% CI: 0.84–1.44); *P* = .49], new onset AF/flutter [7.5% vs 6.7% OR: 1.13 (95% CI: 0.76–1.7); *P* = .54], and VT/VF events [8.2% vs 7.5% OR: 1.11 (95% CI: 0.78–1.57); *P* = .57]. Body mass index reduction was significantly greater in the GLP-1RA group (−6.7% vs −0.82%).

### Falsification outcomes

In the matched cohorts, 112 patients in the GLP-1RA group and 98 patients in the non–GLP-1RA group developed GI bleeding, hip/radial fracture, or a UTI over the follow-up period (OR: 1.16; 95% CI: 0.87–1.54; *P* = .31).

## Conclusions

In the present study, we report that GLP-1RA use was associated with improved outcomes in patients with HFrEF. Moreover, GLP-1RA use was not associated with increased risk of either atrial or ventricular arrhythmias. The safety and clinical utility of GLP-1RAs in this patient population remains uncertain, with conflicting evidence. To date, only two dedicated trials (FIGHT^4^ and LIVE^5^) compared GLP-1RAs to placebo in patients with HFrEF; both trials were relatively small (*n* = 300 and *n* = 241), and used the older drug liraglutide. A recent meta-analysis suggested that GLP-1RAs (vs placebo) were associated with a higher risk of worsening HF events in the HFrEF population (*P* = .046).^7^ However, in the SELECT trial, a prespecified analysis based on investigator-reported baseline diagnosis of HFrEF (*n* = 1347) demonstrated that treatment with semaglutide improved outcomes, including MACE and a composite HF endpoint.^1,8^ Similarly, GLP-1RA significantly reduced cardiovascular mortality in an observational analysis of Swedish patients with LVEF ≤40% and BMI >30 kg/m^2^, with 98% having type 2 DM.^9^ Our study adds to these prior real-world data by also suggesting a potential benefit of GLP-1RA in HFrEF patients. Recent data are also conflicting, with some suggesting that use of GLP-1RAs in HFrEF was associated with a numeric increase in VT/VF events (13 vs 2; *P* = .07) and a significant increase in nonsustained ventricular events and shocks, and anti-tachycardia pacing therapies (33 vs 3; *P* = .01),^10^ while others reported the opposite .^11^ We did not find an association of GLP-1RA use and ventricular dysrhythmias. Nevertheless, these considerations add another layer of complexity in evaluating the benefits of GLP-1RA use in HFrEF.^12^

Our study has multiple limitations. Data were obtained from an aggregate electronic health record database (TriNetX). As such, the accuracy of reported health conditions and the completeness of captured outcomes occurring outside of the database may be limited; hence, potential selection bias from unmeasured confounding factors remains a limitation. However, we conducted robust PSM to strengthen our findings. Another limitation is the inability to confirm consistent adherence to prescribed medications throughout the study period, nor to account for GLP-1RA prescriptions issued by non-participating HCOs. Moreover, the reliance on deidentified, aggregate data from TriNetX limits access to individual patient data, precluding the treatment of GLP-1RA use as a time-varying variable. However, the significantly greater weight loss in the GLP-1RA group over 1 year suggests appropriate classification and ongoing medication use. Cardiovascular mortality is not available within the TriNetX database and, therefore, could not be evaluated in this analysis. Additionally, our 1-year follow-up may be insufficient to detect differences in atherosclerotic endpoints such as ACS, as cardiovascular outcome trials demonstrating MACE reductions with GLP-1RAs typically had follow-up periods of 2 years or more. Rates of SGLT2i (48%) and MRA (43%) in our cohort were suboptimal relative to guideline recommendations, though consistent with contemporary real-world registries.^13^ Finally, as with any retrospective analysis, these results should be interpreted with caution and considered hypothesis-generating rather than practice-changing.

We found that GLP-1RA use in patients with HFrEF was associated with lower risks of all-cause mortality and ADHF without an apparent increase in arrhythmic events. While these findings support the potential therapeutic value of GLP-1RAs in this population, they remain observational. Large, prospective, randomized controlled trials are warranted to assess the safety and efficacy of GLP-1RAs in HFrEF.

## Declarations

### Data Availability

No data were generated or analysed for this manuscript.
